# Synthesis and Application of LTA Zeolite for the Removal of Inorganic and Organic Hazardous Substances from Water: A Review

**DOI:** 10.3390/molecules30030554

**Published:** 2025-01-25

**Authors:** Junyao Pan, Binyu Wang, Simiao Liu, Shanshan Liu, Wenfu Yan

**Affiliations:** State Key Laboratory of Inorganic Synthesis and Preparative Chemistry, College of Chemistry, Jilin University, Changchun 130012, China; pjy23@mails.jlu.edu.cn (J.P.); wangbinyu@ciomp.ac.cn (B.W.); 15143706097@163.com (S.L.); 15713854388@163.com (S.L.)

**Keywords:** **LTA** zeolite, solid waste, water treatment, heavy metal, ion-exchange selectivity order

## Abstract

Industrialization and human activities have caused significant environmental challenges, with water pollution posing severe risks to human health. This underscores the urgent need for effective water treatment solutions. Zeolites, known for their high specific surface area and stability, have gained increasing attention as adsorbents for water treatment. Among zeolites, **LTA** varieties stand out due to their low Si/Al ratio, which enhances ion-exchange capacity, and their cost-effectiveness. This review focuses on the synthesis of low-silica **LTA** zeolites, particularly zeolite A, using natural materials and solid wastes without relying on organic-structure-directing agents (OSDAs). Common pretreatment processes for such synthesis are also highlighted. The review further explores the applications of **LTA** zeolites in water treatment, emphasizing their exceptional performance in adsorbing inorganic and organic pollutants. In particular, **LTA** zeolites are highly effective at removing inorganic cation pollutants through ion exchange. An updated ion-exchange selectivity order, based on previous studies, is provided to support these findings. Overall, this review aims to guide future research and development in water treatment technologies.

## 1. Introduction

As industrialization intensifies and human civilization advances, pollution from human activities has become widespread. Among various forms of pollution, water contamination is particularly concerning due to its direct impacts on ecosystems and human health [1,2,3,4,5,6,7]. Industrial processes, such as metal plating, metallurgy, petroleum refining, battery production, mining, textiles, and pharmaceuticals, generate waste that is often discharged into groundwater, rivers, and lakes, posing significant risks [8,9,10]. Water pollutants include heavy metal ions (e.g., lead, cadmium, copper, zinc, and nickel), harmful anions (e.g., arsenate, chromate, fluoride, and phosphate), organic dyes, and pharmaceutical compounds [11,12]. This underscores the urgent need for effective wastewater treatment methods to meet safety standards and restore contaminated water systems.

The diverse nature of water pollutants poses significant challenges for effective treatment. Conventional methods such as membrane filtration, chemical oxidation, solvent extraction, adsorption, coagulation, flocculation, and biological treatment have notable limitations [13,14,15,16,17,18,19,20]. Many of these techniques rely heavily on chemicals, including chlorides, ammonia, permanganate, alum, sodium hydroxide, hydrochloric acid, ozone, and iron salts. Additionally, they require complex mechanical processes, substantial engineering expertise, and advanced infrastructure [21]. Tailoring individual treatments for each pollutant is often impractical, as traditional technologies are frequently unable to completely remove toxins, phosphorus, nitrogen, and heavy metals [22,23]. In contrast, adsorption offers a simpler, cost-effective, and efficient approach that avoids generating secondary pollution [24]. However, a key challenge in adsorption is the development of adsorbents with high capacity, rapid kinetics, superior selectivity, and excellent recyclability [25].

**Figure 1:**
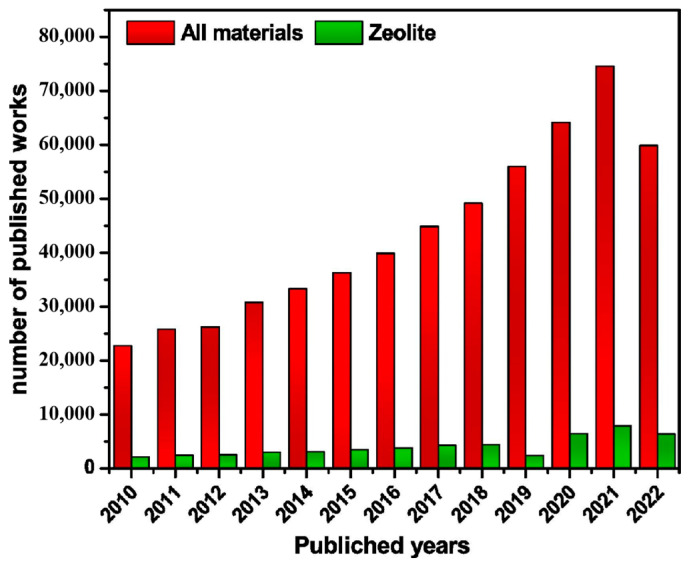
Evolution of the number of works published on the adsorption of wastewater pollutants. Reprinted with permission from Ref. [35]. Copyright (2024) Elsevier.

Zeolites, inorganic porous aluminosilicates, are particularly well-suited for wastewater treatment due to their outstanding thermal and chemical stability. Their high specific surface area, tunable pore structures, and abundant ion-exchange sites enable the efficient removal of toxic substances, including heavy metal ions, from water [25,26,27,28,29]. Both natural and synthetic zeolites have been extensively studied and proven effective for water pollutant adsorption [30,31,32,33,34]. Since 2010, research on zeolite-based adsorbents has grown significantly, emphasizing their potential in mitigating water pollution (Figure 1) [35]. Although the overall number of publications on wastewater adsorption declined between 2022 and 2024, studies focusing on zeolite-based adsorbents consistently accounted for approximately 9% of the total research output.

The Structural Committee of the International Zeolite Association (IZA-SC) currently recognizes over 260 zeolite structures [36]. Among these, zeolite A (Linde type A or **LTA**) is notable as the first commercially synthetized zeolite and is widely used as a gas adsorbent and ion exchanger [37]. While many **LTA** zeolites have been developed with Si/Al ratios (SAR) greater than 1 [38,39,40,41,42,43], OSDA-free zeolite A remains a popular choice for water treatment. Its low Si/Al ratio (SAR = 1) provides abundant [AlO_4_]^-^ sites for ion exchange, resulting in a high adsorption capacity [44]. Additionally, the three-dimensional channels in **LTA** zeolites facilitate rapid ion migration, enabling fast adsorption kinetics. Due to its simple, low-cost, and efficient synthesis, zeolite A remains a leading material for water treatment applications.

This review examines research published since 1995, with a focus on studies from 2010 onward, regarding the synthesis and application of zeolite A in water treatment. We highlight works that emphasize novel synthesis methods or materials demonstrating exceptional water treatment performance. Specifically, we explore advancements in low-cost, environmentally friendly production methods using natural resources and solid waste. The applications of zeolite A and its composites for adsorbing hazardous substances in aqueous solutions are also discussed. An updated ion-exchange selectivity order is provided, encompassing all inorganic cations that can exchange into zeolite A—an aspect not covered in previous reviews. Finally, we present perspectives on the future of zeolite-based water treatment, aiming to guide further research and development. This review serves as a valuable resource for researchers and professionals working in the field of water treatment.

## 2. Zeolite A and Its Synthesis

**Figure 2:**
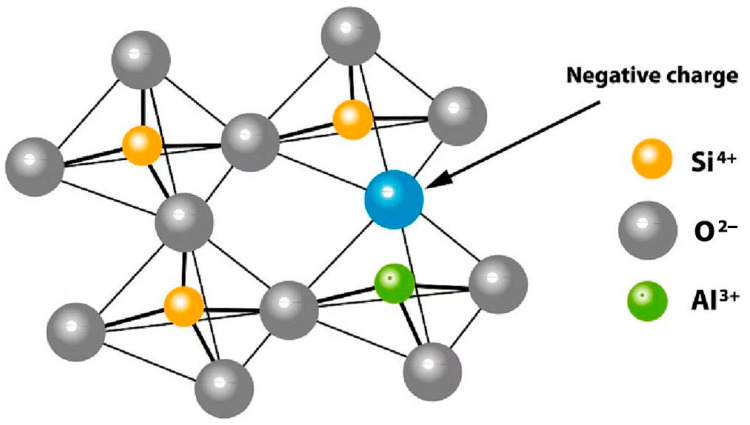
Structure of zeolite **LTA** (source: International Zeolite Association (IZA)). Reprinted with permission from Ref. [45]. Copyright (2023) Elsevier.

Zeolites were first identified in volcanic rocks by Swedish mineralogist Axel Fredrik Cronstedt, who observed that they released water vapor upon heating, resembling boiling. This phenomenon inspired the name “zeolite”. Zeolites are three-dimensional frameworks with uniform, molecular-sized pores, often referred to as molecular sieves. Their structure consists of interconnected TO_4_ tetrahedra (where T represents Si or Al), linked by oxygen atoms. The negative charge from [AlO_4_]^−^ units in the framework is balanced by positive cations located within the pores (Figure 2) [45]. The general empirical formula for zeolites is M_x/n_[(AlO_2_)_x_(SiO_2_)_y_]⋅wH_2_O, where M is an extra-framework cation with valence n, y/x represents the Si/Al ratio, and w denotes the number of water molecules.

Since the 1950s, zeolites have been widely used in various applications. Natural zeolites, formed by the interaction of volcanic rocks and ash with alkaline groundwater, are often impure and therefore rarely used commercially. In contrast, synthetic zeolites are extensively employed in water treatment, gas separation, and catalysis. They serve as ion exchangers, adsorbents, and solid acid catalysts, demonstrating versatility and effectiveness across multiple industries [46,47,48].

**Figure 3:**
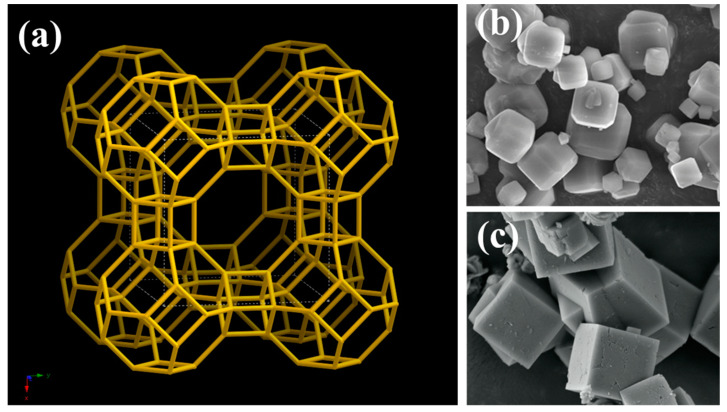
(**a**) Structure of zeolite **LTA** (source: International Zeolite Association (IZA)). (**b**,**c**) SEM images of zeolite A. Reprinted with permission from Refs. [52,53]. Copyright (2018) MDPI and (2024) MDPI.

Zeolite A was the first synthetic zeolite to be commercialized [49]. In its Na^+^-exchanged form, it has the formula Na_12_[(AlO_2_)_12_(SiO_2_)_12_]·27H_2_O, with an Si/Al ratio of 1—the lowest among aluminosilicate zeolites. The Na^+^ ions within the structure are readily exchangeable [50]. The framework of zeolite A consists of two types of cages: *α*-cages and *β*-cages (also known as sodalite cages). Sodalite cages are connected through double four-membered rings (D4Rs), creating a three-dimensional network [37]. Eight sodalite cages fused by D4Rs form a larger *α*-supercage (Figure 3a). The type of cation present (e.g., K^+^, Na^+^, or Ca^2+^) influences the average channel size, leading to different zeolite designations: 3A, 4A, and 5A, respectively [51]. Zeolite A crystals typically exhibit a sharp-edged cubic morphology, as illustrated in Figure 3b,c.

Commercially, zeolite A is synthesized with an Si/Al ratio of approximately 1, resulting in a high density of cation-exchange sites. This property makes it a highly effective adsorbent for water pollution treatment.

### 2.1. Synthesis of Low-Silica LTA from Pure Chemical Raw Materials

Zeolite A can be synthesized using various silicon and aluminum sources through different processes [54,55,56,57,58]. Typically, the synthesis involves mixing an alkaline solution of silicon and aluminum sources to form aluminosilicate gels, followed by hydrothermal treatment at approximately 100 °C to crystallize zeolite A. Key parameters—such as temperature, crystallization time, pH, mixing sequence, and raw material type—significantly influence the synthesis outcome [59,60,61].

For example, Wang et al. studied the effects of inorganic anions on zeolite A synthesis [62]. They observed that Na_2_CO_3_ increased the dissolution rate of zeolite A but had a minimal impact on sodalite crystallization. Na_3_PO_4_ inhibited sodalite crystallization, while Na_2_SO_4_ promoted sodalite formation and improved particle size. Notably, SO_4_^2−^ containing sodalite had a larger unit cell compared to other sodalite types. These findings indicate that CO_3_^2−^ and SO_4_^2−^ destabilize **LTA** phases, whereas PO_4_^3−^ has a stabilizing effect.

Efforts to reduce crystallization temperatures and times have shown promising results. Jafari et al. synthesized zeolite A at 60 °C using aluminum isopropoxide, colloidal silica, and sodium hydroxide [63]. Similarly, Huang et al. produced single crystals and nanocrystal aggregates near room temperature [64]. Zhang et al. synthesized zeolite A at room temperature using various silicon sources (e.g., fumed silica, water glass) and aluminum sources (e.g., aluminum isopropoxide, aluminum powder), though this extended the crystallization times significantly [65]. Sun et al. improved this process, achieving zeolite A in 4 h using atmospheric reflux [66]. Furthermore, advanced techniques such as microwave and ultrasound methods have been used to accelerate crystallization [67,68,69].

Crystal size plays a crucial factor in the performance of zeolite A. Sharma et al. synthesized thermally stable nanocrystals through hydrothermal treatment of ammonium aluminate and sodium silicate at 100 °C for 24 h, with the resulting crystals maintaining structural integrity up to 1000 °C [70]. Fang et al. created submicron zeolite A on a honeycomb ceramic matrix using microwave hydrothermal methods [71]. Mintova et al. demonstrated that ageing amorphous gels for 3 d before hydrothermal treatment enhanced crystal nucleation and growth [72].

Factors such as alkalinity, hydrothermal conditions, and ageing also affect crystal size. Yu et al. found that higher sodium concentrations resulted in larger crystals, while prolonged ageing reduced crystal size and improved uniformity [73]. Bayati et al. reported that higher crystallization temperatures and extended times generally produced larger particles, with temperature having a more significant impact than time [74].

To address diffusion limitations within microporous **LTA** channels, researchers have developed hierarchical zeolite A structures with mesoporosity [75,76,77,78,79]. For example, Chen et al. used three-dimensionally ordered mesoporous carbon as a template to create mesoporous zeolite A [80]. Cho et al. introduced organosilane surfactants into the synthesis mixture to form mesopores [81]. Hasan et al. utilized cetyltrimethylammonium bromide to produce hierarchical zeolite A and synthesized core–shell structures with hollow morphologies through a two-step crystallization process [82,83].

For practical applications, zeolite A often requires granulation to improve mechanical strength. However, traditional binders can reduce its specific surface area and porosity [84,85]. To address this, binder-free microspheres have been developed. Yu et al. created zeolite A/chitosan hybrid microspheres using biomolecules [86], while others incorporated clay during hydrothermal synthesis to produce binder-free zeolite A [87].

### 2.2. Synthesis of Low-Silica LTA from Natural Substances and Solid Waste

**Figure 4:**
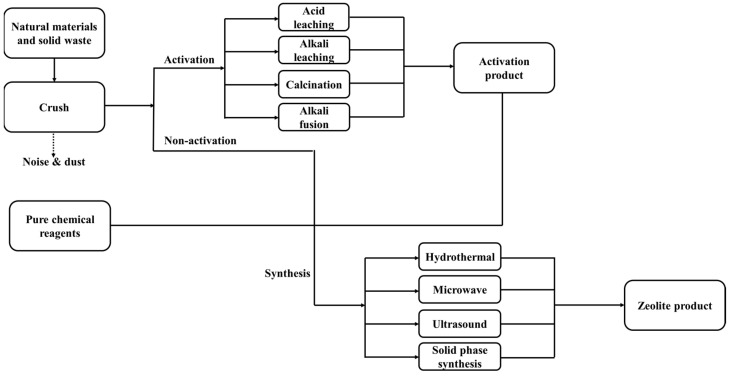
Flow chart of zeolite synthesis from natural materials and solid waste.

Research on synthesizing zeolite A from natural substances and solid waste is primarily conducted at the laboratory scale. The industrial process involves two main stages: raw material activation and product crystallization (Figure 4). Activation methods include high-temperature roasting, acid treatment, and high-temperature solid-phase alkali fusion. The choice of activation method depends on the raw material composition and is tailored accordingly [88,89,90,91].

#### 2.2.1. Coal Fly Ash (CFA)

Coal fly ash (CFA) is generated during coal combustion at temperatures between 1300 and 1500 °C, producing small spherical particles with smooth surfaces and fine micropores [92]. Power plants produce vast amounts of fly ash, exceeding 800 million tonnes annually, with the largest contributions from China, India, and the USA. CFA is a valuable source of silicon, containing high silicon content along with smaller amounts of aluminum, iron, calcium, magnesium, and potassium [93,94]. Despite its potential, a significant portion of CFA is still treated as waste and remains underutilized [95,96].

Research on zeolite synthesis from CFA highlights the importance of the pretreatment process, such as alkali fusion activation. In some cases, acid leaching is also required to extract active Si and Al while minimizing interference from impurities like Fe and mullite [97,98]. For instance, Cao et al. synthesized zeolite A by calcining CFA at 750 °C for 3 h [99]. Similarly, Fan et al. used CFA calcined at 700 °C for 3 h to prepare zeolite A [100]. Yang et al. investigated the effects of NaOH and Na_2_CO_3_ on CFA activation for synthesizing zeolite 4A [101]. They found that using Na_2_CO_3_ alone produced zeolite 4A with slightly higher crystallinity but required a higher alkali dosage. However, mixing Na_2_CO_3_ and NaOH in a 2.8:1 mass ratio yielded zeolite 4A with the highest crystallinity (75.8%) under conditions of low energy and water consumption. In other studies, Nowak et al. synthesized zeolite A from low-quality CFA and wet flue gas desulphurization wastewater [102], while Kunecki et al. optimized activation conditions to 550 °C for 1 h [103].

In addition to solid-phase activation, liquid-phase methods have been developed for synthesizing zeolite A from CFA. These methods typically require milder conditions, making them more cost-effective and safer. For example, Chareonpanich et al. activated CFA by dispersing it in sodium hydroxide solution, heating it at 120 °C for 4 h, and synthesizing zeolite A in an integrated process [104]. This approach simplifies operations by reducing the need for specialized equipment, such as Teflon-lined autoclaves. Due to variations in coal composition from different sources, tailored preparation strategies are often necessary for synthesizing zeolite **LTA** [37].

#### 2.2.2. Kaolin and Clay

Kaolin clay (Al_2_Si_2_O_3_(OH)_4_) is a significant source of silica and alumina for the synthesis of zeolite A. Numerous studies have employed kaolin as a feedstock for zeolite A production [105,106,107]. When calcined at temperatures between 550 and 900 °C, kaolin transforms into reactive metakaolin [108].

Holmes et al. calcined Nigerian kaolin at 650 °C for 10 min to produce metakaolin. However, the sample retained quartz, which limited the quality of the synthesized zeolite A despite attempts to remove impurities [109]. In contrast, Schwanke et al. successfully synthesized antibacterial zeolite A using Brazilian Amazon kaolin mining waste. The waste was calcined at 600 °C for 2 h to form metakaolin [110]. Wang et al. synthesized flaky zeolite NaA with kaolin as the sole source of Si and Al, achieving a specific surface area of 39.8 m^2^·g^−1^, mainly mesoporous. This was significantly higher than that of the surface area of traditional NaA [111].

Efforts to simplify and create milder pretreatment methods for zeolite synthesis have increased. Wang et al. proposed an approach that bypasses calcination by directly reacting natural kaolin with NaOH to hydrothermally [112]. This forms hydrous sodium aluminosilicates, which are then dissolved in dilute HCl to yield an amorphous Si-Al gel. The gel is suitable for hydrothermal synthesis of zeolite A. Compared to conventional calcination methods, this process offers better control over particle size and morphology, resulting in enhanced brightness and cation-exchange capacity.

Microwave-assisted synthesis has emerged as an alternative to traditional hydrothermal methods, as it has improved energy conduction, leading to smaller and more uniform zeolites [113]. Oliveira et al. converted metakaolin into zeolite A using microwave-assisted techniques, achieving excellent CO_2_ adsorption capacity [114]. Similarly, Chen et al. observed that microwave radiation reduced activation energy and accelerated crystallization during kaolin calcination and 4A zeolite synthesis [115].

Solvent-free methods have also gained attention. Yang et al. developed a solvent-free strategy to prepare self-supporting hierarchical porous zeolites, using polymethyl methacrylate (PMMA) microspheres as sacrificial templates [116].

Beyond kaolin, other natural clays can also serve as effective raw materials for zeolite A synthesis. Si et al. synthesized zeolite A from spherical clay calcined at 600 °C and crystallized at 60 °C for 48 h [117]. García et al. also prepared zeolite A with excellent optical properties using natural clay [118]. Foroughi et al. explored the effects of clay type on product morphology during zeolite A synthesis [119]. Zeolite A synthesized from a clay mixture containing illite, kaolinite, and pyrophyllite exhibited semi-cubic particles with rounded edges and a lower surface area (17.5 m^2^·g^−1^). In contrast, products synthesized from kaolinite-rich clay were mesoporous, with an average pore size of 8 nm and a larger surface area (~59.6 m^2^·g^−1^).

#### 2.2.3. Coal Gangue

Coal gangue (CG), a hazardous by-product of coal mining, constitutes approximately 10–15% of raw coal production. Its massive accumulation presents significant environmental challenges worldwide [120]. However, CG is rich in SiO_2_ and Al_2_O_3_, making it a valuable resource for zeolite synthesis and an environmentally friendly application for waste management.

Several studies have explored methods for synthesizing zeolite A from CG. Qian et al. and Wang et al. adopted an in situ synthesis method, which involved calcining CG for decarburization, followed by melting with NaOH at 400–500 °C for 2 h [44,121]. Liu et al. developed a two-step synthesis process to produce high-whiteness zeolite A. This approach included high-temperature calcinations to reduce Fe_2_O_3_ content, followed by hydrothermal acid leaching to remove Fe impurities and enhance ion-exchange capacity [122]. Similarly, Kong et al. utilized a pretreatment of calcination, acid leaching and alkali fusion to synthesize zeolite A from high-iron, high-quartz CG [123].

Recently, simplified methods have emerged that omit some pretreatment steps beyond calcination, streamlining the zeolite A synthesis process from CG [124,125,126].

#### 2.2.4. Rice Husk Ash and Other Biomass Ash

Rice husk ash (RHA), a by-product of rice processing, contains more than 90% silica, which can exist in crystalline or amorphous forms depending on combustion conditions [127,128]. Its low cost and wide availability make RHA an attractive raw material for zeolite A synthesis.

The typical synthesis process involves calcining rice husk at approximately 700 °C to produce RHA. This ash is then activated with sodium hydroxide to extract silica as a soluble silica sol. Acid leaching with HCl or HNO_3_ may be used to precipitate impurities, yielding a purified silica gel for zeolite synthesis [129,130,131]. For example, Madhu et al. synthesized zeolite A with high CO_2_ adsorption performance by extracting SiO_2_ from RHA with NaOH [132]. Ghasemi et al. enhanced the process by using HCl to remove impurities prior calcination [133].

Schmitz et al. investigated the impact of different RHA sources on zeolite A synthesis. They found that the proportion of amorphous silica in RHA significantly influences its solubility in NaOH, which affects the crystallization process [129]. Amorphous silica obtained through controlled calcination typically produces larger crystals and better intergrowth of cubic crystals compared to commercial fumed silica. Tan et al. examined how calcination temperature and duration affect RHA composition and zeolite morphology [134]. They observed that RHA calcined at higher temperatures and for longer durations develops a porous structure, enhancing solubility and reaction efficiency. Nawog et al. simplified the process by synthesizing nano-NaA zeolites directly from RHA without silica extraction or the use of OSDAs, reducing solvent and reagent consumption [135].

Other biomass ash sources, such as sugarcane bagasse ash [136], barley husk [137], wheat husk [138], date leaf midribs [139], phragmites australis [140], and bamboo leaf [141], have also been explored for zeolite A synthesis. For instance, Küçük et al. utilized biomass fly ash from a co-incineration plant at a paper mill to synthesize zeolite A [142]. Azizi et al. synthesized zeolite A from barley husk, using NaOH pretreatment to extract silica [143]. Moisés et al. optimized silica utilization by combining sugarcane bagasse ash with solid NaOH, melting the mixture at 550 °C and subsequently adding water to prepare a sodium silicate solution for zeolite A synthesis [144].

#### 2.2.5. Diatomite

Diatomite, also known as diatomaceous earth, is a sedimentary rock composed of fossilized single-celled algae [145]. Its primary component is amorphous silica or hydrated opaline silica, valued for its chemical stability and inertness. Diatomite is widely used in filtration and adsorption applications, such as managing organic waste in the food industry [146,147]. Recycling diatomite into value-added products offers both environmental and economic benefits.

Recent studies have explored diatomite as a raw material for zeolite synthesis. For example, Nascimento et al. synthesized zeolite A from diatomite with a silicon–aluminum ratio of approximately 1–2 [148]. El-Kordy et al. developed zeolite A–clay composites using diatomite combined with natural clay [149].

#### 2.2.6. Mineral Waste Residue

Mineral waste residue, generated during mining and mineral extraction, can pose environmental hazards if improperly managed. However, slag containing high levels of Si or Al offers a cost-effective and sustainable option for zeolite synthesis.

Li et al. synthesized highly crystalline zeolite A from calcined lithium slag at a low temperature of 60 °C [150]. Wu et al. utilized calcined and HCl-leached opal waste, achieving zeolite A with excellent ammonium exchange capacity [151]. Lei et al. activated bauxite tailings via alkali fusion at 500 °C for 3 h to produce zeolite A [152]. Yan et al. created magnetic zeolite A from red mud and coal gangue using an alkali-reducing calcination-hydrothermal method [153]. Kuroki et al. prepared zeolite A by reacting crushed stone powder with aluminum ash following alkali and acid treatments [154].

#### 2.2.7. Waste Glass and Sand

Waste glass, commonly sourced from beverage bottles, windscreens, and television panels, is often recycled. However, conventional recycling methods require heating to around 1500 °C, leading to high energy consumption and significant greenhouse gas emissions [155].

Yao et al. synthesized zeolite A from waste cathode ray tubes, examining the effects of hydrothermal temperature and the SiO_2_/Al_2_O_3_ ratio on the final product [156]. At lower temperatures (80–100 °C) and pressure (0.47–1.01 bar), and at an SiO_2_/Al_2_O_3_ ratio of 2.0, amorphous phases were observed. Increasing the temperature to 110 °C and pressure to 1.43 bar yielded NaA zeolite, along with NaP1 and Faujasite. Higher temperatures and pressures caused NaA and Faujasite to disappear, with hydroxysodalite becoming the dominant phase. The SiO_2_/Al_2_O_3_ ratio also influenced the phases formed: a ratio of 1.5 produced pure NaA, while a ratio of 2.0 led to a mixture of NaA and Faujasite. Lee et al. used waste glass from liquid crystal displays (LCDs) and sandblasting waste to synthesize zeolite A, optimizing the process to achieve superior water adsorption and desorption properties [157]. Similarly, Prasertsab et al. synthesized zeolites **LTA** and **FAU** from waste sand generated during oil and gas production [158].

#### 2.2.8. Aluminum Waste

Aluminum is an essential metal in various industries, including aerospace, automotive, food and beverage, construction, and electrical/electronics. Consequently, large amounts of aluminum waste are generated, particularly from beverage cans, highlighting the need for sustainable recycling and reuse methods.

Terzano et al. synthesized zeolite A from municipal glass and aluminum wastes using a one-pot process without prior activation [155]. Tounsi et al. prepared zeolite A by reacting sodium silicate, extracted from Tunisian sand, with aluminum scrap dissolved in NaOH [159]. Similarly, Abdelrahman dissolved aluminum cans in NaOH, filtered out impurities, and synthesized zeolite A using various silicon sources, including fumed silica, sodium metasilicate, silica sol, and tetraethyl orthosilicate [160].

#### 2.2.9. Furnace Slag

Furnace slag is a by-product of the iron smelting industry and is primarily composed of Si, with notable amounts of Al, Ca, Mg, and other metals [161].

Sugano and Murakami attempted to synthesize zeolite A directly from blast furnace slag, but the resulting product had low purity, with only 60% zeolite A content [162,163]. They identified that the presence of Ca and Mg oxides hindered zeolite A crystallization. To address this, Anuwattana proposed a method involving calcination followed by acid pickling to remove these impurities, improving the synthesis of zeolite A [164]. Similarly, Li et al. used co-calcination of blast furnace slag with NaOH to produce zeolite A [165].

Linz–Donawitz (LD) slag is generated during the conversion of pig iron to crude steel in the Linz–Donawitz process [166]. Samanta et al. synthesized zeolite A from LD slag and evaluated its ability to remove Fe^3+^ ions and the cationic dye methylene blue, as discussed in the next section [167,168]. The activation process for LD slag included calcination, acid pickling, and alkali fusion activation.

#### 2.2.10. Natural Zeolites

Natural zeolites have been used for centuries, but their impurities and low crystallinity limit their industrial applications [169,170]. Despite this, the high Si and Al content of natural zeolites, along with their zeolite phases, has attracted significant interest in synthesizing more effective synthetic zeolites from these materials.

For example, Faghihian et al. synthesized zeolite A from natural clinoptilolite through an interzeolite transformation method after refluxing the material for 3 days [171,172]. Similarly, Kazemian et al. transformed natural clinoptilolite into zeolite A without the need for activation [173]. Yue et al. produced high-purity zeolite A from natural stellerite sourced from Gongxi, China, by co-calcination with Na_2_CO_3_ at temperatures between 700 and 900 °C [174].

#### 2.2.11. Spent FCC Catalysts

The fluid catalytic cracking (FCC) process is essential in petroleum refineries, particularly for gasoline production [175]. Catalysts facilitate the cracking of hydrocarbons to yield valuable products. However, this process generates coke, which deposits on the catalyst’s active sites, leading to deactivation. Additionally, metals such as vanadium (V), iron (Fe), and nickel (Ni) can accumulate on the catalyst surface, further contributing to deactivation [176].

The FCC process generates substantial amounts of spent catalysts, which mainly consist of ZSM-5, zeolite Y, and various alumina substrates. These spent catalysts may also contain toxic metals like V, Fe, and Ni, as well as rare earth metals such as lanthanum (La) and cerium (Ce). Gonzalez et al. synthesized zeolite A from waste FCC catalysts without activation, although the conversion efficiency of the raw materials needed improvement [177]. They later proposed a high-temperature alkali fusion activation method to enhance conversion [178]. Monzón et al. employed a similar method and achieved zeolite A with good crystallinity [179].

To reduce energy consumption and improve safety, Wang et al. introduced a low-temperature (200 °C) alkali fusion activation method for synthesizing zeolite A (designated as SFCC-A) from waste FCC catalysts [33]. The resulting SFCC-A exhibited crystallinity and ion adsorption capacities comparable to commercial zeolite A.

#### 2.2.12. Waste Ceramics

The ceramic industry generates significant amounts of waste ceramics, primarily in the form of silicon-based materials, which can potentially be converted into zeolites [180]. This conversion offers an opportunity to recycle waste ceramics, contributing to sustainability and resource efficiency within the industry.

Wajima et al. developed a method to extract silica from waste ceramics using NaOH solution, subsequently synthesizing zeolite A from the recovered silica [181]. The silica recovery rate achieved was an impressive 26,000 mg/L. This approach has since been adopted by other researchers to convert waste ceramics into zeolite A [182].

#### 2.2.13. Electrolytic Manganese Residue

Electrolytic manganese residue (EMR) is a by-product generated during the solid-liquid separation step in the acid leaching of manganese ore to prepare electrolytes. EMR presents significant environmental challenges and incurs high treatment costs, limiting the sustainable development of manganese metallurgy.

The components of EMR vary depending on their source but typically consist predominantly of silica [183]. Li et al. utilized EMR to synthesize zeolite A for the adsorption of inorganic cationic pollutants [184,185,186]. Their process involved alkali fusion activation at 800 °C, followed by ageing the product in deionized water. The activated material was filtered, and the resulting filtrate was used for hydrothermal synthesis.

#### 2.2.14. Other Raw Materials

In addition to the materials mentioned above, several natural and waste-derived substances have been explored as precursors for zeolite A synthesis. These include natural materials such as perlite [187], volcanic ash [188], halloysite [189,190], and oil shale [191]. Solid waste sources, such as waste incineration fly ash [192,193], red mud [194], waste peat ash [195], coal gasification slag [196], tannery wastewater [197], and foundry dust [198], have also been effectively utilized.

### 2.3. Synthesis of High/Pure Silica LTA

**Figure 5:**
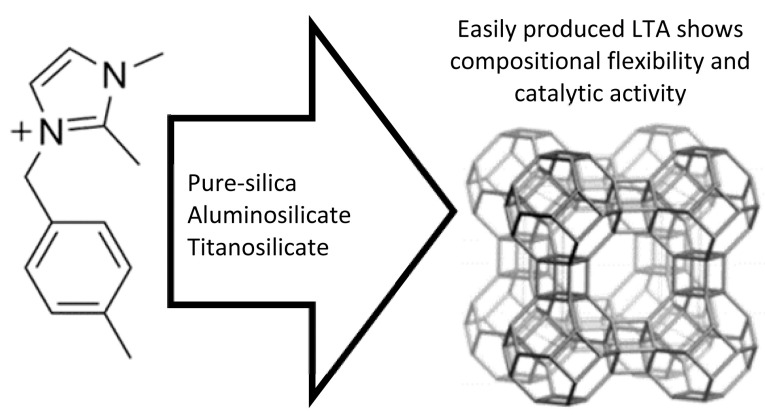
Imidazolium OSDA for the synthesis of ITQ-29 and high-silica **LTA** zeolites. Reprinted with permission from Ref. [200]. Copyright (2015) ACS.

The first pure silica **LTA** zeolite, known as ITQ-29, was synthesized using fluoride media in conjunction with methylated julolidine and tetramethylammonium (TMA^+^) as OSDAs [199]. Since then, efforts have focused on developing ITQ-29 using alternative OSDAs. Tiscornia et al. synthesized ITQ-29 membranes on alumina tubular supports using a supramolecular molecule along with TMA^+^ [42]. Boal et al. introduced a novel imidazolium-based OSDA, which, when used combined with TMA^+^ as a co-OSDA, optimized the synthesis of ITQ-29 [200]. Their method also enabled the production of high-silica and silicogermanium variants of **LTA** zeolites with Si/Al ratios ranging from 12 to 42. Imidazolium-based ITQ-29 seeds were employed to achieve these results (Figure 5). This strategy has since been widely adopted for synthesizing high-silica **LTA** zeolites.

Previously, zeolite A with an Si/Al ratio greater than 1 (known as ZK-4) was typically synthesized using TMAOH as the OSDA in potassium (K^+^)-mediated systems [38,39,40,43,201,202]. However, these methods rarely produced zeolites with Si/Al ratios exceeding 3. Tao et al. successfully synthesized high-silica **LTA** zeolites with an Si/Al ratio of 6 by using tetramethylammonium, tetraethylammonium, and dimethyldiethylammonium as co-OSDAs [203]. The introduction of imidazolium-based OSDAs further advanced the synthesis of **LTA** zeolites, enabling Si/Al ratios of 10 or even higher. Hong’s group leveraged this approach to synthesize a series of high-silica **LTA** zeolites for applications such as NH_3_-SCR [204,205,206,207,208].

### 2.4. Synthesis of AlPO/SAPO LTA

The zeolite A variant AlPO/SAPO is also known as AlPO-42/SAPO-42. Its synthesis typically requires a fluoride (F^−^) introduction system, as reported in recent studies [209,210,211,212]. Similar to the synthesis of high/pure silica **LTA** and silicogermanium **LTA**, aluminophosphate-based **LTA** can be synthesized using TMA^+^ and rigid imidazole-based molecules as OSDAs.

In 2010, Huang et al. synthesized large single crystals of AlPO-LTA using the crown ether Kryptofix 222 as an OSDA [213]. Azim et al. employed 1-butylpiperidine chloride in an ionothermal method to produce AlPO-42 [214]. Xu et al. used n-propylamine as an OSDA to hydrothermally synthesize large single crystals of AlPO-42 [215]. Lin et al. utilized 1-ethyl-3-methyl imidazolium bromide with tetraalkylammonium ionic liquid in ionothermal synthesis [210]. AlPO-42 has also been synthesized using co-OSDAs, including 1-butyl-3-methylimidazolium bromide and TMA^+^ as co-OSDAs [209,211,212].

For SAPO-42, Pinilla-Herrero et al. demonstrated the use of TMA^+^ and diethanolamine (DEA) as co-OSDAs [216,217]. Lin et al. and Vinaches et al. synthesized SAPO-42 using imidazole-based OSDAs under ionothermal and hydrothermal conditions, respectively [218,219]. Additionally, Martínez-Franco et al. developed two self-assembled aromatic molecules—2,2-dimethyl-2,3-dihydro-1H-benzo[de]isoquinoline-2-ium and 4-methyl-2,3,6,7-tetrahydro-1H,5H-pyrido[3.2.1-ij]quinolinium as OSDA for SAPO-42 synthesis. These strategies pave the way for synthesizing aluminophosphate-based and high-silicon **LTA** [220].

## 3. Removal of Hazardous Substances from Water by Zeolite A

Zeolite A and its composites have been widely studied for removing hazardous substances from water. These studies primarily target inorganic cation pollutants, including toxic heavy metals, simulated radionuclides, water-softening ions such as Mg^2+^ and Ca^2+^, and ammonia nitrogen. Some research also addresses the removal of organic pollutants, such as dyes and pharmaceuticals. Zeolite A demonstrates high adsorption capacities for most metal cations, as summarized in Table 1. For example, Sr^2+^ adsorption capacity reaches 294.1 mg·g^−1^, while Pb^2+^ achieves 880 mg·g^−1^. The adsorption of cationic pollutants is generally described by the Langmuir isotherm and pseudo-second-order kinetic models, highlighting ion exchange as the primary mechanism. In modified zeolite A, alternative models such as the Friedrich isotherm and pseudo-first-order kinetics may apply due to structural changes affecting adsorption behavior.

### 3.1. Removal of Heavy Metal Cations

The rapid pace of industrialization, coupled with human activities such as mining and waste generation, has led to a significant increase in heavy metal pollution. Industrial waste containing heavy metals contaminates the environment, posing severe health risks. Once heavy metals enter the human body, they can inhibit enzyme activity, induce cytotoxicity, damage nerve tissues, and impair detoxification organs [221,222,223].

Low-silica zeolite A is an excellent material for addressing heavy metal contamination due to its high specific surface area and abundant cation-exchange sites. Several studies have explored its synthesis and application.

Cui et al. utilized a small amount of TMA^+^ as an OSDA to synthesize low-silica zeolite A [224]. By adjusting the ratio of inorganic to organic structure-directing agents, they achieved a low Si/Al ratio, high crystallinity, reduced crystal size, and enhanced adsorption performance. Under optimal conditions—90 min of contact time, pH 5, 25 °C, and adsorbent dosage of 100 mg·g^−1^—the maximum adsorption capacities were 230 mg·g^−1^ for Cu(II) and 600 mg·g^−1^ for Pb(II).

Nseke et al. synthesized zeolite 4A from waste materials, including silicon sources and aluminum foil [225]. They optimized adsorption parameters for Cu^2+^, Zn^2+^, and Cd^2+^, achieving capacities of 99.9 mg·g^−1^, 82.1 mg·g^−1^, and 103 mg·g^−1^, respectively. Key conditions included a zeolite dosage of 12 g·L^−1^, pH 6, a contact time of 1–3 h, and a temperature of 25–27.5 °C.

Mubarak et al. synthesized zeolite 4A from kaolin and modified it with TiO_2_ [226]. The TiO_2_@Zeolite-4A exhibited faster adsorption kinetics and higher removal efficiency for Fe(III) and Mn(II) at lower pH levels compared to unmodified zeolite.

Ghasemi et al. used microwave-assisted methods to prepare zeolite 4A for Ni^2+^ removal [227]. The removal efficiency exceeded 99% under optimized conditions, with adsorption following the Langmuir isotherm model. The maximum capacity increased from 94.3 mg·g^−1^ at 298 K to 185.2 mg·g^−1^ at 333 K.

Meng et al. synthesized zeolite A from halloysite and evaluated its selectivity for heavy metal ions [189]. The material showed the highest selectivity for Pb^2+^ and Ag^+^, with capacities of 227.7 mg·g^−1^ and 123.0 mg·g^−1^, respectively. The selectivity order was Ag^+^ ~ Pb^2+^ > Cr^3+^~Cu^2+^~Zn^2+^ > Mn^2+^~Ni^2+^~Fe^3+^.

Rentsennorov et al. synthesized zeolite A from fly ash and reported an adsorption capacity of 35.8 mg·g^−1^ for Cr(III) [228]. The material reached equilibrium within 30 min, achieving 97% removal efficiency for a 10 mg/L Cr(III) solution. Xiao et al. synthesized zeolite A from CFA for Cr(VI) removal, reporting a lower capacity of 8.7 mg·g^−1^ [229]. To enhance Cr(VI) adsorption, Guan et al. modified zeolite 4A with water-soluble chitosan quaternary ammonium salt (HACC), achieving a capacity of 16.9 mg·g^−1^ [230].

Li et al. developed an environmentally friendly method for Hg(II) removal using NaA zeolite synthesized from CFA [231]. Zn^2+^ and S^2−^ ions were introduced into the zeolite channels via ion exchange to form ZnS nanoclusters. During Hg^2+^ removal, Hg^2+^ formed HgS compounds by exchanging with Zn^2+^ in the ZnS-zeolite structure. This process prevented Zn^2+^ leaching and secondary pollution. The ZnS-zeolite NaA composite exhibited an impressive Hg^2+^ adsorption capacity of 553.2 mg·g^−1^ and maintained over 99.9% removal efficiency in the presence of Pb^2+^, Cd^2+^, and Cu^2+^, with only a 2% reduction in efficiency after five cycles, demonstrating its strong potential for practical applications.

### 3.2. Removal of Radionuclide Cations

Radionuclide cations such as ^60^Co^2+^, ^90^Sr^2+^, and ^137^Cs^+^, are commonly found in wastewater generated from smelting, petrochemical processing, nuclear operations, and mineral mining. The Fukushima nuclear accident heightened concerns about safely treating nuclear fission products, particularly ^137^Cs and ^90^Sr. Similar to other metal cations, these radionuclides can be effectively removed using zeolite A via ion exchange. In laboratory studies, non-radioactive cations are often used as proxies to simulate the adsorption behavior of radioactive counterparts.

Fang et al. compared the removal efficiencies of ^137^Cs^+^, ^90^Sr^2+^, and ^60^Co^2+^ using zeolite 4A, natural zeolite, and vermiculite in laundry wastewater containing organic materials and suspended solids [232]. Zeolite 4A achieved the highest removal efficiencies, exceeding 90% for all three radionuclides under neutral conditions at room temperature and surpassing 98.7% under alkaline conditions.

Murukutti et al. synthesized zeolite A from CFA and evaluated its adsorption capacities for ^137^Cs^+^ and ^90^Sr^2+^ [233]. The adsorption capacities were 95.7 mg·g^−1^ for ^137^Cs^+^ and 54.1 mg·g^−1^ for ^90^Sr^2+^.

Lee et al. investigated the removal of ^137^Cs using zeolite A [234]. Synchrotron X-ray diffraction studies revealed that as ion exchange progressed, Cs^+^ ions shifted to occupy the centers of eight-membered rings (8MRs). **RHO** zeolite, which contains more 8MRs than zeolite A, exhibited superior adsorption performance for Cs^+^, confirming the role of 8MRs in enhancing selectivity.

Wang et al. scaled up the synthesis of high-quality zeolite A (SFCC-A) from spent fluid catalytic cracking (FCC) catalysts to a 100 L batch [33]. SFCC-A exhibited an adsorption capacity of 180.5 mg·g^−1^ for Co^2+^ and achieved over 99% removal efficiency at a pH range of 4-8 and a solid-to-liquid ratio of 1/1000. Its performance was comparable to that of commercial zeolite A, highlighting its potential for industrial applications.

Hao et al. studied the removal of ^90^Sr^2+^ using NaA zeolite [235]. Adsorption capacity reached 294.1 mg·g^−1^ under optimized conditions, with a distribution coefficient K_d_ of 421.56 ± 13.39 L·g^−1^. In radioactive wastewater containing high concentrations of NaNO_3_ and NH_4_NO_3_, NaA zeolite maintained stable performance, reducing radioactive concentrations of 0.62 ± 0.12 Bq·L^−1^, near regulatory limits for drinking water. Structural Rietveld refinements showed that Sr^2+^ occupied the center of the single six-membered rings (s6rs), while density functional theory (DFT) calculations confirmed high selectivity for ^90^Sr^2+^ in eight-membered rings (s8rs). In related work, Wang et al. synthesized zeolite A from lithium slag, achieving a high adsorption capacity of 246.9 mg·g^−1^ for Sr^2+^ with equilibrium reached within 5 min [32]. The material demonstrated effective performance across a wide pH range (4–13), confirming its potential for radioactive wastewater treatment.

Dahake et al. developed polyamidoxime-modified zeolite A for uranium adsorption [236]. The modified zeolite achieved a 98% removal efficiency for [UO_2_]^2+^, with an adsorption capacity of 4.6 mg U·g^−1^. Even in the presence of competitive ions such as Cr, Cd, Co, Pb, and Mn, or after eight adsorption cycles, the removal efficiency remained above 90%.

### 3.3. Water Softening

Ca^2+^ and Mg^2+^ ions are the primary contributors to water hardness. Hard water causes scaling in machines, boilers, and pipelines. It has also been linked to health issues, including cardiovascular disease, kidney stones, and certain cancers. Moreover, hard water reduces soap efficiency, increasing detergent consumption [237,238].

Kusrini et al. evaluated acid-activated zeolite A, synthesized from kaolin, for water softening [239]. At an initial concentration of 110 ppm and a solution volume of 20 mL, 1 g of zeolite A removed 96.0% of Ca^2+^. Increasing the dosage to 2 g improved Mg^2+^ removal efficiency to 94.9%.

Manna et al. synthesized zeolite A from CFA and red mud, achieving adsorption capacities of 184.6 mg·g^−1^ for Ca^2+^ within 15 min and 253.9 mg·g^−1^ for Mg^2+^ after 5 h [240]. Similarly, El-Nahas et al. produced low-cost zeolite A from waste silica gel and aluminum. Their zeolites removed over 90% of total hardness (>1000 ppm) within 30 min [241].

Xue et al. developed meso-zeolite A with intracrystalline mesopores (~3 nm diameter) [242]. These mesopores significantly reduced the activation energy for ion exchange, accelerating Mg^2+^ diffusion. The hydrated Mg^2+^ diffusion in meso-zeolite A was 17.5 times higher than in conventional zeolite A at 273 K.

Painer et al. synthesized zeolite A from perlite and tested it for water softening [243]. Their zeolite removed 99.8% of Ca^2+^ and 93.4% of Mg^2+^ from tap water, outperforming both **SOD** zeolite and perlite powder. These findings underscore the potential of zeolite A for water softening applications.

### 3.4. Removal of Ammonia-Nitrogen

Ammonia-nitrogen, a non-metallic cationic pollutant, is commonly found in freshwater systems due to agricultural fertilizers and domestic sewage [244]. Elevated levels contribute to eutrophication, oxygen depletion, and toxicity to aquatic life [245]. Additionally, ammonia-nitrogen can corrode metals and pose indirect health risks when converted by nitrifying bacteria into nitrite and nitrate, which are harmful in drinking water [246].

Numerous studies have demonstrated the potential of zeolite A (**LTA**) for ammonia-nitrogen removal. Wang et al. synthesized zeolite A from foundry dust, achieving an adsorption capacity of 37.8 mg·g^−1^, which decreased to 28.6 mg·g^−1^ after four regeneration cycles [198]. Ren et al. used CFA to produce zeolite A, reporting an NH_4_^+^ adsorption capacity of 2.322 mmol·g^−1^ (41.8 mg·g^−1^) [247]. Similarly, Jiang et al. synthesized zeolite A from CFA with an NH_4_^+^ adsorption capacity of 41.2 mg·g^−1^ [248]. In both studies, the adsorption process followed pseudo-second-order kinetics and the Langmuir model. Zhao et al. synthesized zeolite NaA from halloysite, achieving an NH_4_^+^ adsorption capacity of 44.3 mg·g^−1^ with excellent regeneration and reusability [190]. Wu et al. produced zeolite 4A from opal waste rock, which exhibited a high NH_4_^+^ adsorption capacity of 53.1 mg·g^−1^ [151]. The adsorption isotherm followed the Freundlich model (R^2^ > 0.99), suggesting multi-layer adsorption was feasible for NH_4_^+^ removal.

### 3.5. Removal of Inorganic Anion Pollutants

Inorganic anions generally exhibit low adsorption efficiency on zeolite frameworks due to charge repulsion between the anions and negatively charged zeolite frameworks. To address this, researchers have modified zeolite A or developed composites to enhance anion removal from water.

For fluoride (F^−^) removal, Wang et al. synthesized zeolite A from coal gangue, achieving a modest adsorption capacity of 4.7 mg·g^−1^ [249]. In contrast, Naskar synthesized zeolite A from rice husk ash, modified it with colloidal hydrated alumina, and achieved an F^−^ adsorption capacity of 104.2 mg·g^−1^, with recyclability up to three cycles [250]. Chakraborty et al. developed a zeolite A-MgO composite, which exhibited a similar F^−^ adsorption capacity of 107.6 mg·g^−1^ and maintained performance for five cycles [251]. The adsorption process in these studies adhered to pseudo-second-order kinetics and the Langmuir model, indicating a monolayer, chemically driven adsorption mechanism.

Phosphate removal has also been investigated extensively. Kugbe et al. used a zeolite A-goethite nanocomposite to achieve a phosphate adsorption capacity of 500 μmol·g^−1^, outperforming the individual components [252]. Guan et al. functionalized zeolite 4A with tetraethylenepentamine, achieving an endothermic adsorption process with a maximum capacity of 28 mg·g^−1^ at 25 °C, following the Langmuir model [253]. Song et al. reported a La-containing magnetic NaA zeolite synthesized from coal gangue, which achieved a phosphate adsorption capacity of 44.6 mg·g^−1^, nine times higher than the precursor zeolite [253].

Arsenic removal has focused on As(III) and As(V) species, which exist as oxygen-containing anions in water. Pillewan et al. demonstrated the use of Cu-exchanged zeolite A for As(III) and As(V) removal, achieving adsorption capacities of 1.4 mg·g^−1^ for As(III) and 1.48 mg·g^−1^ for As(V) [254]. Abukhadra et al. developed a chitosan/zeolite A hybrid structure with an As(V) adsorption capacity of 125 mg·g^−1^. The adsorption behavior followed pseudo-second-order kinetics and the Freundlich isotherm models, indicating multi-layer adsorption driven by chemical interactions [255].

Other studies have explored the removal of additional anions. Ashraf et al. evaluated a cellulose fibers/zeolite A nanocomposite for selenium species removal, achieving adsorption capacities of 163 mg·g^−1^ for selenite, 212.4 mg·g^−1^ for selenite, and 109.3 mg·g^−1^ for selenomethionine [256]. The adsorption followed pseudo-first-order kinetics and the Langmuir model, indicating homogenous, monolayer adsorption.

### 3.6. Removal of Organic Pollutants

Zeolite A has been extensively studied for removing organic pollutants, including dyes and pharmaceuticals. However, the large size of many organic molecules often prevents their penetration into the zeolite channels, limiting adsorption to the surface. To address this limitation, researchers have modified zeolite A to enhance its adsorption capacity for organic pollutant removal.

Haghjoo et al. synthesized zeolite A from CFA and modified it with hexadecyltrimethylammonium chloride (HDTMAC) [257]. The modified zeolite exhibited a glyphosate pesticide adsorption capacity of 769.2 mg·g^−1^ and a removal efficiency of 98.9%, demonstrating its potential for sewage purification.

Djioko et al. synthesized zeolite A from kaolin for ciprofloxacin removal, achieving an adsorption capacity of 87.7 mg·g^−1^ [258]. Using 20 mg of adsorbent in a 60 ppm ciprofloxacin solution, the removal efficiency reached 91.8% at 25 °C. Even after seven cycles, the removal efficiency remained high at 85.7%. The adsorption process followed pseudo-second-order kinetic and the Langmuir model, indicating a monolayer chemical adsorption mechanism.

Mostafa et al. prepared a chitosan/zeolite A (CS/ZA) composite to evaluate its performance against organophosphorus pesticides, including acephate, omthosate, and methyl parathion [259]. The adsorption capacities were 650.7 mg·g^−1^ for acephate, 506.5 mg·g^−1^ for omthosate, and 560.8 mg·g^−1^ for methyl parathion. In fixed-bed column systems (6 cm) with a flow rate of 5 mL·min^−1^ and pH 8, removal efficiencies were 78%, 57.6%, and 74.3%, respectively. Unlike most zeolite adsorbents, the adsorption followed a pseudo-first-order kinetic model, suggesting physical adsorption as the dominant mechanism.

Modified zeolite A has also been explored for dye removal. Muniandy et al. used NaOH-modified 3A zeolite to remove malachite green and methyl violet dyes, achieving adsorption capacities of 136 mg·g^−1^ and 186 mg·g^−1^, respectively [260]. Xu et al. synthesized zeolite A from CFA and achieved a maximum adsorption capacity of 40.6 mg·g^−1^ for acid fuchsin [261].

Nyankson et al. developed a zeolite A/Fe_3_O_4_ nanocomposite for methylene blue removal, achieving a Langmuir adsorption capacity of 2.6 mg·g^−1^ at 25 °C and a maximum adsorption efficiency of 97.5% [262]. After regeneration, the efficiency at pH 7 decreased slightly to 82.6%. Similarly, Elfeky et al. demonstrated that ZnO-modified zeolite A enhanced methylene blue removal efficiency from 67.8% to 94.8% [263].

Khalaf et al. investigated zeolite A modified with hexadecyltrimethylammonium bromide for Congo red removal [264]. Batch experiments showed a maximum removal efficiency of 99.2% after 90 min, with an adsorption capacity of 21.1 mg·g^−1^.

**Table 1:** Adsorption performance towards hazardous substances by zeolite A and zeolite A-based composites.

Raw Material	Adsorbents	Pollutant	Adsorption Capacity	Removal Efficiency	Kinetics	Adsorption Isotherm	Ref.
Lithium slag	Zeolite A	Sr(II)	246.9 mg·g^−1^	99.9%	pseudo-second-order	Langmuir	[32]
PCR *	Zeolite A	Sr(II)	294.1 mg·g^−1^	>99%	pseudo-second-order	Langmuir	[235]
PCR	Zeolite A	Sr(II)	204.3 mg·g-^1^	> 80%	pseudo-second-order	-	[265]
PCR	Zeolite A	Sr(II)	5.4 meq·g^−1^	83%	-	-	[266]
Kaolinite and red mud	Magnetic zeolite A	Sr(II)	172 mg·g^−1^	96.4%	pseudo-second-order	Langmuir	[267]
PCR	magnetic zeolite A	Cs(I) Sr(II)	229.3 mg·g^−1^ 89.0 mg·g^−1^	81.4% 95.2%	pseudo-second-order	Langmuir	[268]
Metakaolin	Zeolite A	Sr(II) Co(II)	167.5 mg·g^−1^ 118.5 mg·g^−1^	-	pseudo-second-order	Langmuir	[269]
Halloysite	Zeolite A	Pb(II) Ag(I)	227.7 mg·g^−1^ 123.0 mg·g^−1^	100%	-	-	[189]
PCR	Zeolite A	Cu(II) Pb(II)	230 mg·g^−1^ 600 mg·g^−1^	-	pseudo-second-order	Langmuir	[224]
Bagasse ash	Zeolite A	Cu(II) Pb(II)	63.2 mg·g^−1^ 187.2 mg·g^−1^	40.2% 37.5%	-	-	[136]
Metakaolin	Magnetic zeolite NaA	Cu(II) Pb(II)	2.3mmol·g^−1^ 2.3mmol·g^−1^	>95% >95%	-	-	[270]
Wheat husk	NaCl-modified **LTA**	Pb(II)	321.8 mg·g^−1^	-	-	-	[138]
BFA and CFA	ZBG ZCF	Pb(II)	625 mg·g^−1^ 556 mg·g^−1^	100%	pseudo-second-order	Langmuir	[271]
CFA	Zeolite A	Pb(II)	714.3 mg·g^−1^	-	first-order	Langmuir	[272]
Metakaolin	Zeolite A	Pb(II)	880 mg·g^−1^	74.5%	pseudo-second-order	Langmuir	[273]
Metakaolin	Zeolite A	Pb(II)	529.7 mg·g^−1^	>99%	pseudo-second-order	Langmuir	[274]
Lithium leach residue	Zeolite A	Pb(II) Cd(II)	487.8 mg·g^−1^ 193.8 mg·g^−1^	100% 96.9%	pseudo-second-order	Langmuir	[275]
Rare earth tailings	Zeolite A	Cd(II) Cu(II) NH_4_^+^ P(V) F^−^	247.3 mg·g^−1^ 137.1 mg·g^−1^ 35 mg·g^−1^ 13.8 mg·g^−1^ 5.9 mg·g^−1^	99.6% 98.2% 70.0% 38.2% 15.4%	pseudo-second-order	Langmuir	[276]
PCR	Zeolite A	Pb(II) Cu(II) Cr(III) Zn(II) Co(II)	400 mg·g^−1^ 396 mg·g^−1^ 391 mg·g^−1^ 385 mg·g^−1^ 393.5 mg·g^−1^	100% 99% 97% 96% 98%	pseudo-second-order	Langmuir	[277]
Perlite	Zeolite A	Eu(III) Ce(III)	6.0 mg·g^−1^ 5.1 mg·g^−1^	99% 90%	-	Langmuir	[278]
Micrometersized **LTA**	Nano **LTA**	Cs(I)	422 mg·g^−1^	45%	pseudo-first-order	Langmuir	[279]
Kaolin	Zeolite A lattices	Cs(I)	106.3 mg·g^−1^	-	pseudo-second-order	Freundlich	[280]
PCR	PAN–zeolite A	Cs(I) Sr(II)	214.1 mg·g^−1^ 98.1 mg·g^−1^	90% 90%	pseudo-second-order	Langmuir and D–R	[281]
PCR	MWCNT @Zeolite A	Cs(I) Sr(II)	113 mg·g^−1^ 107 mg·g^−1^	-	pseudo-second-order	Langmuir	[282]
CFA	Zeolite A	Cs(I) Sr(II)	2.1 mmol·g^−1^ 1.9 mmol·g^−1^	-	pseudo-second-order	Langmuir	[283]
CFA	Zeolite A	Cr(III)	35.8 mg·g^−1^	97%	-	Langmuir	[228]
Bauxite tailings	Zeolite A	Cr(III)	85.1 mg·g^−1^	96.8%	-	-	[152]
CFA	Zeolite A	Cr(VI)	8.7 mg·g^−1^	-	-	-	[229]
PCR	4A/HACC	Cr(VI)	16.9 mg·g^−1^	92%	pseudo-second-order	Langmuir	[230]
Kaolin	Zeolite A	Cr(VI)	9.7 mg·g^−1^	100%	-	-	[284]
PCR	Zeolite A/ Fe_3_O_4_/biochar	Cr(VI)	46.9 mg·g^−1^	93.9%	pseudo-second-order	Langmuir	[285]
PCR	Zeolite A	Cr(III)	70 mg·g^−1^	-	pseudo-second-order	-	[197]
Kaolin	Zeolite A	Cr(III)	~200 mg·g^−1^	99.8%	pseudo-second-order	-	[286]
PCR	m-ZPC	Cu(II) Cr(III)	3.9 mg·g^−1^ 2.0 mg·g^−1^	-	pseudo-second-order	Redlich–Peterson	[287]
Waste materials	Zeolite A	Cd(II) Cu(II) Zn(II)	103 mg·g^−1^ 99.9 mg·g^−1^ 82.1 mg·g^−1^	96% 98~99.9% 80~85%	-	Freundlich and Langmuir	[225]
Woody biomass ash	Zeolite A	Cu(II) Cd(II) Pb(II)	140.1 mg·g^−1^ 223.5 mg·g^−1^ 850.7 mg·g^−1^	>99%	pseudo-second-order	Freundlich and Langmuir	[142]
CFA	Zeolite A	Ni(II) Cd(II) Pb(II)	1.1 mmol·g^−1^ 1.4 mmol·g^−1^ 2.6 mmol·g^−1^	-	pseudo-second order	Langmuir	[288]
PCR	Hierarchical **LTA**	Pb(II) Cu(II) Ni(II)	510 mg·g^−1^ 170 mg·g^−1^ 100 mg·g^−1^	-	pseudo-second-order	Langmuir	[25]
Red mud	Magnetic 4A-zeolite	Zn(II) Cu(II) Cd(II) Ni(II) Pb(II)	45.4 mg·g^−1^ 35.6 mg·g^−1^ 56.5 mg·g^−1^ 41.2 mg·g^−1^ 100.0 mg·g^−1^	-	pseudo-second-order	Langmuir	[194]
Red mud and coal gangue	Magnetic zeolite A	Cu(II) Cd(II) Pb(II)	76.2 mg·g^−1^ 92.2 mg·g^−1^ 178.4 mg·g^−1^	-	pseudo-first-order pseudo-second-order pseudo-second-order	Langmuir	[153]
Rice husk and waste aluminum cans	Geopolymer/ zeolite A	Co(II) Cu(II) Zn(II)	127.2 mg·g^−1^ 119.1 mg·g^−1^ 121.8 mg·g^−1^	-	pseudo-second order	Langmuir	[289]
Red mud and CGS	Magnetic zeolite A	Pb(II) Cu(II)	330.7 mg·g^−1^ 142.7 mg·g^−1^	85%	pseudo-second-order Elovich	Langmuir Freundlich	[196]
Kaolin	O_2_-plasma treatment Zeolite A	Cd(II)	247.0 mg·g^−1^	71%	-	-	[290]
PCR	hierarchical **LTA**	Cd(II)	324.3 mg·g^−1^	90%	pseudo-first-order	Langmuir	[291]
CFA	Zeolite A	Pb(II) Cd(II)	277.8 mg·g^−1^ 87.7 mg·g^−1^	-	pseudo-second order	Langmuir	[292]
PCR	Zeolite A	Zn(II)	117.4 mg·g^−1^	79.6%	pseudo-second-order	Langmuir	[293]
PCR	Zeolite A	Zn(II) Cd(II)	4.0 mmol·g^−1^ 2.0 mmol·g^−1^	-	pseudo-second-order	Freundlich and D-R	[294]
PCR	Zeolite A	Cu(II)	202.8 mg·g^−1^	70.3%	pseudo-first-order	Langmuir	[295]
PCR	Fe_3_O_4_@zeolite NaA	Cu(II)	86.5 mg·g^−1^	86.5%	pseudo-second-order	Langmuir	[296]
PCR	magnetic zeolite A	Cu(II)	170 mg·g^−1^	57%	-	Langmuir	[297]
PCR	Zeolite A	Cu(II)	155.4 mg·g^−1^	93.3%	-	-	[298]
Coal gangue and aluminum ash	Zeolite A	Cu(II)	75.0 mg·g^−1^	99%	pseudo-second-order	-	[121]
Coal gangue	ZMC ZTC ZAC	Cu(II)	118.1 mg·g^−1^ 116.7 mg·g^−1^ 116.1 mg·g^−1^	-	pseudo-second-order	Langmuir	[125]
PCR	Hierarchical **LTA**	Cu(II)	341.5 mg·g^−1^	-	pseudo-second-order	Freundlich	[299]
Low-grade bauxite	Zeolite A	Cd(II)	161.3 mg·g^−1^	99.9%	pseudo-second-order	Langmuir and Freundlich	[300]
PCR	tGO-Zeo	Cd(II)	196 mg·g^−1^	-	-	Langmuir	[301]
Metakaolin	Zeolite A	Cu(II)	698.1 mg·g^−1^	-	pseudo-second-order	Langmuir	[302]
SFCC	Zeolite A	Co(II)	180.5 mg·g^−1^	99.2%	pseudo-second-order	Langmuir	[33]
Agricultural waste	AAS AWS	Co(II)	235.2 mg·g^−1^ 202.9 mg·g^−1^	-	-	Freundlich	[303]
PCR	Zeolite A	Ni(II)	94 mg·g^−1^~(25 °C) 132 mg·g^−1^~(45 °C) 185 mg·g^−1^~(60 °C)	99.9%	pseudo-second-order	Langmuir	[227]
PCR	NaA/XG -alginate	Co(II) Ni(II)	43.9 mg·g^−1^ 81.3 mg·g^−1^	-	pseudo-second-order	Langmuir	[304]
Rice husk and waste aluminum cans	geopolymer/ zeolite A/ chitosan	Hg(II) Pb(II)	211.9 mg·g^−1^ 269.5 mg·g^−1^	-	pseudo-second-order	Langmuir	[305]
CFA	NH_3_ modified zeolite 4A	Hg(II)	53.6 mg·g^−1^	99.2%	pseudo-second-order	Langmuir	[306]
CFA	ZnS-zeolite NaA	Hg(II)	553.2 mg·g^−1^	>99.9%	pseudo-second-order	Langmuir and Freundlich	[231]
PCR	C@zeolite-ZnS	Hg(II)	795.8 mg·g^−1^	99.9%	pseudo-second-order	Langmuir	[307]
Opal	Zeolite A	Hg(II)	42.0 mg·g^−1^	70%	pseudo-second order	Langmuir	[308]
Commercial	Zeolite A	Th(IV)	2.8 meq·g^−1^	50.4%	-	Langmuir	[309]
PCR	Na_2_SO_4_@ zeolite A with MnO_2_	^226^Ra ^228^Ra	-	78.7% 66.7%	-	-	[310]
PCR	Co-Zn-LTA	Tc(VII)	-	88.3%	pseudo-second-order	-	[311]
PCR	Zeolite A	U(VI)	0.95 mg·g^−1^	60-67%	pseudo-first-order	Langmuir	[312]
PCR	Zeolite A	U(VI)	1.08 mg·g^−1^	>96%	pseudo-first-order	Langmuir	[313]
Kaolin	TiO2@ Zeolites-4A	Fe(III) Mn(II)	150.1 mg·g^−1^ 94.1 mg·g^−1^	94% 100%	pseudo-second-order	Freundlich and Langmuir	[226]
PCR	Agarose- Zeolite **LTA**	Al(III) Mn(II) Fe(III)	15.8 mg·g^−1^ 3.0 mg·g^−1^ 19.2 mg·g^−1^	99.5% 95.6% 95.3%	-	-	[314]
Linz–Donawitz (LD) slag	Zeolite A	Fe(III)	27.6 mg·g^−1^	99.9%	pseudo-second-order	Langmuir	[167]
PCR	Zeolite A	Mn(II)	30 mg·g^−1^~(25 °C) 50 mg·g^−1^~(55 °C)	~70%	-	Langmuir	[315]
Kaolinite	ZC ZF	Mn(II)	6.8 mg·g^−1^ 7.2 mg·g^−1^	82.2% 99.9%	pseudo-second-order	Langmuir	[316]
Electrolytic manganese residue	Zeolite A	Mn(II) Cd(II)	119.5 mg·g^−1^ 314.2 mg·g^−1^	- 85.6%	pseudo-second-order	Langmuir	[184,185]
PCR	Cu-LTA	As(III) As(V)	1.4 mg·g^−1^ 1.5 mg·g^−1^	>98%	pseudo-first-order	Langmuir	[254]
CFA	NZVI-5A	As(V)	72.1 mg·g^−1^	84.0%	pseudo-second-order	Langmuir	[317]
PCR	Fe-HZ	As(V)	5.1 mg·g^−1^	-	pseudo-second-order	Langmuir	[318]
PCR	Zeolite 5A	As(V) Pb(II)	36.4 mg·g^−1^ 46.7 mg·g^−1^	>95%	Elovich model	Langmuir and Freundlich	[319]
CFA and red mud	Zeolite A	Ca(II) Mg(II)	184.6 mg·g^−1^ 253.9 mg·g^−1^	-	pseudo-second-order	-	[240]
Waste aluminum and silica gel	Zeolite A	Ca(II) Mg(II)	111.1 mg·g^−1^ for Ca(II)	90%	pseudo-second-order intraparticle diffusion	Freundlich and Temkin	[241]
PCR	Mesoporous **LTA**	Ca(II) Mg(II)	3.1 mmol·g^−1^ 2.8 mmol·g^−1^	-	pseudo-second-order	Dual-site Langmuir	[242]
Metakaolin	Zeolite A	Ca(II) Mg(II)	935 mg·g^−1^	>94%	pseudo-second-order	Langmuir	[320]
Kaolin	Acid-activated zeolite A	Ca(II) Mg(II)	-	95.9% 94.9%	-	-	[239]
Perlite	Zeolite A	Ca(II) Mg(II)	2.7 mmol·g^−1^	99.8% 93.4%	-	-	[243]
PCR	Zeolite A	Cs(I) Sr(II) Ca(II) Mg(II)	1.6 mmol·g^−1^ 5.5 mmol·g^−1^ 4.8 mmol·g^−1^ 4.2 mmol·g^−1^	-	-	Dubinin–Radushkevich	[321]
CFA	Zeolite A	Ca(II)	184 mg·g^−1^	-	-	-	[322]
PCR	CTAB modified Zeolite A	Ca(II)	129.3 mg·g^−1^	95.0%	pseudo-second-order	D–A and Langmuir	[323]
Bauxite tailings	Zeolite A	Ca(II)	296 mg·g^−1^	38.4%	-	-	[324]
Coal gangue	Zeolite A	Ca(II)	296.0 mg CaCO3·g^−1^	-	-	-	[122]
Coal gangue	Zeolite A	Ca(II)	358 mg·g^−1^	-	-	-	[44]
PCR	Polyamidoxime-modified zeolite A	U(VI)	4.9 mg·g^−1^	98%	pseudo-second-order	Langmuir	[236]
PCR	Chitosan/ zeolite A	Cd(II) As(V)	170 mg·g^−1^ 125 mg·g^−1^	100%	pseudo-first-order pseudo-second-order	Freundlich	[255]
PCR	Nanomagnetite/**LTA**	Dy(III)	35 mg·g^−1^	100%	pseudo-second-order	Langmuir and Temkin	[325]
PCR	NaA membrane	MoO_4_^2-^	-	99.8%	-	-	[326]
PCR	Pt/**LTA**	Ba(II) La(III)	30.0 mg·g^−1^ 14.8 mg·g^−1^	99.9% 99.9%	pseudo-second-order	Freundlich Langmuir	[327]
PCR	Tetraethylenepentamine- modified zeolite 4A	P(V)	23 mg·g^−1^	-	pseudo-second-order	Langmuir	[253]
PCR	Zeolite A– goethite	P(V)	0.5 mmol·g^−1^	-	-	Langmuir	[252]
PCR	Fe-LTA	P(V)	5.8 mg·g^−1^	80%	pseudo-second-order	Langmuir	[328]
Coal gangue	LMZ	P(V)	44.6 mg·g^−1^	99.6%	-	-	[126]
Kaolin	Co_3_O_4_@ zeolite@ nano SiO_2_	P(V)	344.8 mg·g^−1^	-	pseudo-first-order	Langmuir	[329]
PCR	CF/ZA	Se(VI) Se(IV) Se(Mt)	163 mg·g^−1^ 212.4 mg·g^−1^ 109.3 mg·g^−1^	100% 100% 79.7%	pseudo-first-order	Langmuir	[256]
Opal waste rock	Zeolite A	NH_4_^+^	53.1 mg·g^−1^	-	-	Freundlich	[151]
PCR	Zeolite A	NH_4_^+^	42.6 mg·g^−1^	-	pseudo-second-order	Langmuir	[330]
PCR	Zeolite A	NH_4_^+^	31.9 mg·g^−1^	-	-	Langmuir	[331]
PCR	Fe_3_O_4_/**LTA**	NH_4_^+^	10.5 mg·g^−1^	84.0%	pseudo-second-order	Freundlich	[332]
Halloysite	Zeolite A	NH_4_^+^	44.3 mg·g^−1^	-	-	Langmuir and Freundlich	[190]
CFA	Zeolite A	NH_4_^+^	41.2 mg·g^−1^	>40%	pseudo-second-order	Langmuir	[248]
CFA	Zeolite A	NH_4_^+^	60.6 mg·g^−1^	59.6%	-	Freundlich	[333]
CFA	Zeolite A	NH_4_^+^	2.3 mmol·g^−1^	-	-	-	[247]
Foundry dust	Zeolite A	NH_4_^+^	37.8 mg·g^−1^	96.2%	pseudo-second-order	Langmuir	[198]
Halloysite	Chitosan/ zeolite A	NH_4_^+^	47.6 mg·g^−1^	-	-	Langmuir	[334]
Kaolin	Zeolite A	NH_4_^+^	122.0 mg·g^−1^	-	pseudo-second-order	Langmuir	[335]
PCR	Zeolite A	NH_4_^+^	73.0 mg·g^−1^	95.8%	pseudo-second-order	Langmuir and Freundlich	[336]
PCR	Zeolite A	NH_4_^+^	94.2 mg N·g^−1^	70.2%	pseudo-second-order	Freundlich	[337]
PCR	Zeolite A	NH_4_^+^	2.6 mmol·g^−1^	-	-	Langmuir	[338]
PCR	Zeolite A-MgO	F^−^	107.6 mg·g^−1^	72%	pseudo-second-order	Langmuir	[251]
Coal gangue	Zeolite A	F^−^	4.7 mg·g^−1^	94.8%	pseudo-second-order	-	[249]
Rice husk ash	Zeolite A	F^−^	104.2 mg·g^−1^	99%	pseudo-second-order	Langmuir	[250]
PCR	Zeolite A/Fe_3_O_4_	Methylene blue	2.6 mg·g^−1^	97.5%	pseudo-second-order	Langmuir	[262]
Linz–Donawitz (LD) slag	Zeolite A	Methylene blue	25.3 mg·g^−1^	98.1%	pseudo-second-order	Langmuir	[168]
PCR	Fe_3_O_4_/ZA	Methylene blue	40.4 mg·g^−1^	∼96.8%	pseudo-second-order	-	[339]
Kaolin	Zeolite A	Methylene blue	44.4 mg·g^−1^	99.4%	pseudo-second-order	Langmuir	[340]
CFA	Zeolite A	Methylene blue	23.2 mg·g^−1^ (25 °C) 43.8 mg·g^−1^ (40 °C)	82.9%	pseudo-second-order	Langmuir	[341]
PCR	NaA_mw_	Methylene blue	64.8 mg·g^−1^	-	pseudo-first-order	Langmuir	[342]
PCR	MZ-A/RGO	Methylene blue Pb(II)	666.7 mg·g^−1^ 416.7 mg·g^−1^	98.5% 93.9%	pseudo-second-order	Langmuir	[343]
PCR	3A zeolite	Methyl violet	136 mg·g^−1^	60%	pseudo-first-order	Langmuir	[260]
PCR	3A zeolite	Malachite green	186 mg·g^−1^	96%	pseudo-first-order	Langmuir	[260]
Waste aluminum cans	Zeolite A	Malachite green	29.7 mg·g^−1^	-	pseudo-second-order	Langmuir	[160]
PCR	HDTMABr modified **LTA**	Congo red	21.1 mg·g^−1^	99.2%	pseudo-second-order	Temkin	[264]
CFA	Zeolite A	Acid fuchsin	40.6 mg·g^−1^	-	pseudo-first-order	Langmuir	[261]
CFA	Surfactant-modified-**LTA**	Glyphosate	769.2 mg·g^−1^	98.9%	pseudo-second-order	Freundlich	[257]
PCR	Cu-LTA	Glyphosate	112.7 mg·g^−1^	-	pseudo-first-order and pseudo-second-order	Langmuir	[339]
Kaolin	Zeolite A	Ciprofloxacin	87.7 mg·g^−1^	91.8%	pseudo-second-order	Langmuir	[258]
PCR	CS/ZA	AC OM MP	650.7 mg·g^−1^ 506.5 mg·g^−1^ 560.8 mg·g^−1^	78% 57.6% 74.3%	pseudo-first-order	Langmuir	[259]
Clay	Zeolite A	Methylene blue 8-HQ	77.1 mg·g^−1^ 33.5 mg·g^−1^	-	-	Freundlich	[344]
PCR	CS/ZA	Bezactive Orange 16	305.8 mg·g^−1^	-	pseudo-second-order	Langmuir	[345]
Coal gangue	ZMC ZTC ZAC	Rh-B	5.4 mg·g^−1^ 13.1 mg·g^−1^ 32.8 mg·g^−1^	-	pseudo-second-order	Langmuir	[125]
PCR	GO/4A	Rh-B	62.8 mg·g^−1^	-	pseudo-second-order	Langmuir	[346]
CFA	Zeolite A	AR 66	416.7 mg·g^−1^	100%	pseudo-second-order	Freundlich	[347]
PCR	CTAB modified Zeolite A	Methyl orange	22.4 mg·g^−1^	87.2%	-	Langmuir	[348]

* PCR refers to pure chemical reagents.

## 4. Ion-Exchange Selectivity Order on Zeolite A

Studies on metal cation removal using zeolite A have demonstrated significant differences in ion-exchange selectivity. These differences manifest in the adsorption capacities of zeolite A for various cations, its preference in mixed cation systems, and the challenges of replacing one metal cation for another. These factors are influenced by the **LTA** framework’s affinity for specific cations and the hydrated ion sizes.

For example, Finish et al. observed that zeolite 4A exhibited a much higher affinity for Pb^2+^ and Cr^3+^ compared to Zn^2+^, Cd^2+^, and Ni^2+^ [349]. Similarly, Ibrahim et al. synthesized zeolite A from Egyptian kaolin and reported the selectivity order as Pb^2+^ > Cd^2+^ > Cu^2+^ > Zn^2+^ > Ni^2+^ [350]. Meng et al. proposed a comparable order: Ag^+^ ≈ Pb^2+^ > Cr^3+^ ≈ Cu^2+^ ≈ Zn^2+^ > Mn^2+^ ≈ Ni^2+^ ≈ Fe^3+^ [189].

Hui et al. synthesized zeolite 4A from CFA and determined the selectivity order as Cu^2+^ > Cr^3+^ > Zn^2+^ > Co^2+^ > Ni^2+^, based on adsorption capacity in mg·g^−1^ [351]. However, when normalized to molar adsorption (mmol·g^−1^), Cr^3+^ exhibited a higher capacity than Cu^2+^.

Hao et al. calculated the interaction energies of Na^+^ within the *s8r* rings of the *lta* cage. They found that replacing all cations with Na^+^ in the *s8r* was thermodynamically favorable (Table 2) [235]. This finding underscores the versatility of zeolite 4A for metal cation removal. Additional thermodynamic simulations and experimental studies reported a selectivity order of Sr^2+^ > Ba^2+^ > Cd^2+^ > Ca^2+^ > Mg^2+^ > Cs^+^ > Na^+^ [321,352,353].

Integrating findings from multiple studies, the overall selectivity order for cations on zeolite A is as follows: Ag^+^ ≈ Pb^2+^ > Sr^2+^ > Ba^2+^ > Cr^3+^ > Cd^2+^ > Cu^2+^ > Zn^2+^ > Co^2+^ > Ni^2+^ ≈ Mn^2+^ ≈ Fe^3+^ > Ca^2+^ > K^+^ > Mg^2+^ > NH_4_^+^ > Cs^+^ > Na^+^. Notably, aside from Pb^2+^, zeolite A’s affinity for most divalent cations is relatively similar, a subject of ongoing debate among researchers [354,355]. In practical applications, especially in multi-component systems, the separation efficiency for divalent cations may not always be distinct. However, the selectivity for monovalent ions—except Ag^+^—is significantly lower. This indicates that divalent cations, such as Cu^2+^, Mn^2+^, and Co^2+^, can be effectively separated from monovalent ions. Furthermore, the low selectivity for Na^+^ enhances the suitability of zeolite A as an adsorbent for removing diverse metal cation pollutants.

## 5. Conclusions and Prospects

Adsorption methods are widely favored in water pollution treatment due to their simplicity, eco-friendliness, and low operation costs. Zeolite A has emerged as a highly effective material, offering broad adsorption capabilities for various pollutants.

This review has summarized recent advancements in the synthesis of **LTA** zeolites, with a focus on producing zeolite A with a low Si/Al ratio from natural materials and industrial solid wastes, without relying on OSDAs. The low Si/Al ratio increases the number of ion-exchange sites, thereby enhancing adsorption capacity. Furthermore, the development of cost-effective and environmentally friendly synthesis methods aligns with the growing demand for sustainable water treatment solutions.

We have also reviewed the current status of zeolite A and its derivatives in adsorbing various water pollutants, highlighting representative results as references for future research. For metal cation adsorption, we have outlined the selectivity order of different cations based on experimental data and findings from multiple studies, providing valuable insights into ion-exchange research.

Future research should focus on the development of low-cost, environmentally friendly zeolite A adsorbents derived from natural materials and industrial wastes, with a focus on enhancing their adsorption efficiency. The established ion-exchange selectivity order can be leveraged to optimize wastewater treatment processes, particularly for industrial and domestic effluents. This approach could also facilitate the selective recovery of valuable metal elements, contributing to circular economy practices and sustainable development.

The synthesis of zeolite A from solid waste has matured significantly, offering great potential for practical applications. Establishing on-site systems for synthesizing zeolite A directly at solid waste generation sites or near polluted water sources could further streamline the process. This integrated approach would convert solid waste into high-value zeolite A, which can then be immediately used to treat pollutants at the source. Such a system would eliminate the need to transport raw materials and adsorbents, reducing costs and improving overall sustainability.

## Figures and Tables

**Table 2:** Interaction energies (in eV) of Na^+^ in the *s8r* of the *lta* cage exchanging with various cations. [235]. Copyright (2022) RSC.

Adsorption Reaction	ΔE (eV)
LTA + 2H_2_O + NH_4_^+^·(H_2_O)_4_ → LTA-NH_4_ + Na^+^·(H_2_O)_6_	−0.65
LTA + K^+^·(H_2_O)_6_ → LTA-K + Na^+^·(H_2_O)_6_	−2.13
LTA + Cs^+^·(H_2_O)_6_ → LTA-Cs + Na^+^·(H_2_O)_6_	−0.11
LTA + 6H_2_O + Mg^2+^·(H_2_O)_6_ → LTA-Mg + 2Na^+^·(H_2_O)_6_	−1.94
LTA + 6H_2_O + Ca^2+^·(H_2_O)_6_ → LTA-Ca + 2Na^+^·(H_2_O)_6_	−6.17
LTA + 6H_2_O + Sr^2+^·(H_2_O)_6_ → LTA-Sr + 2Na^+^·(H_2_O)_6_	−8.17

## Data Availability

No new data were created or analyzed in this study. Data sharing is not applicable to this article.
